# The Use of Protein-Protein Interactions for the Analysis of the Associations between PM2.5 and Some Diseases

**DOI:** 10.1155/2016/4895476

**Published:** 2016-05-08

**Authors:** Qing Zhang, Pei-Wei Zhang, Yu-Dong Cai

**Affiliations:** ^1^School of Life Sciences, Shanghai University, Shanghai 200444, China; ^2^Institute of Health Sciences, Shanghai Institutes for Biological Sciences, Chinese Academy of Sciences, Shanghai 200031, China

## Abstract

Nowadays, pollution levels are rapidly increasing all over the world. One of the most important pollutants is PM2.5. It is known that the pollution environment may cause several problems, such as greenhouse effect and acid rain. Among them, the most important problem is that pollutants can induce a number of serious diseases. Some studies have reported that PM2.5 is an important etiologic factor for lung cancer. In this study, we extensively investigate the associations between PM2.5 and 22 disease classes recommended by Goh et al., such as respiratory diseases, cardiovascular diseases, and gastrointestinal diseases. The protein-protein interactions were used to measure the linkage between disease genes and genes that have been reported to be modulated by PM2.5. The results suggest that some diseases, such as diseases related to ear, nose, and throat and gastrointestinal, nutritional, renal, and cardiovascular diseases, are influenced by PM2.5 and some evidences were provided to confirm our results. For example, a total of 18 genes related to cardiovascular diseases are identified to be closely related to PM2.5, and cardiovascular disease relevant gene DSP is significantly related to PM2.5 gene JUP.

## 1. Introduction

Though air pollution varies widely depending on its regions, average pollution levels are increasing rapidly around the world, especially in some industrializing countries in Asia. With the effect of weather and seasons, regional hazes might appear due to the mixture of pollutants, which further lead to visibility impairment, traffic jams, and the reducing of living qualities [1]. Currently, measurement of PM2.5 (particulate matter with particle aerodynamic diameters of 2.5 *μ*m and smaller, also called fine particulate matter) is the most used method as an indicator pollutant to monitor air quality [2]. The sources of PM2.5 are diverse but mostly are from industrial emissions, biomass burning, domestic heating, and cigarette smoking. Also, the annual average range of haze can differ from 10 to 100 *μ*g/m^3^ globally [1].

Exaggerated by air pollution, the increasing of health risks, such as lung diseases, cardiovascular diseases, and inflammation, is threatening us [3]. The influenced degree towards our health is arguable. Until now, our methods to understand how PM2.5 can influence our health are limited. This leads to the situation that most researches mainly focus on lung diseases and ignore other health risks we are facing. Undoubtedly, in this way, our health risks will be largely underestimated.

In order to comprehensively understand our risks, we turn our attention to the associations between PM2.5 and most human disorders. Since diseases are so countless that we hardly calculate each one's relationship with PM2.5, we chose 22 disorder classes recommended by Goh et al. [4], which include most known human disorders and are mainly classified based on the physiological system affected. The relevant genes of each disorder, retrieved from Online Mendelian Inheritance in Man (OMIM) [5], and genes modulated by PM2.5, reported in a study authored by Gualtieri et al. [6], were employed in mining the possible associations between the investigated disorders and PM2.5. For each disease class, we calculated the maximum interaction score of each related gene in this class to PM2.5 relevant genes to evaluate its impact caused by PM2.5 based on the protein-protein interactions (PPIs) from STRING (Search Tool for the Retrieval of Interacting Genes/Proteins) [7]. In addition, a permutation test was executed for each related gene to further evaluate the accuracy of the aforementioned measurement, yielding another measurement, namely, permutation FDR. Finally, for each disease class, the proportion of genes with permutation FDRs less than 0.05 was calculated, which was used to evaluate the strength of the associations between the disease class and PM2.5. Our results show that five disease classes are highly related to PM2.5: ear, nose, throat, gastrointestinal, nutritional, renal, and cardiovascular. These results recovered some known associations between PM2.5 and some diseases, validating the accuracy of the results and providing a new way to investigate the associations between PM2.5 and other diseases.

## 2. Materials and Methods

### 2.1. Genes Related to 22 Diseases

The genes for each disease were downloaded from OMIM (http://www.omim.org/, accessed in January 2014) [5]. Because there are too many diseases and many diseases are actually similar, according to the morbid map file with OMIM disease ID and class assignment proposed in Goh et al.'s study [4], similar diseases were combined into the same disease class. 22 disease classes were obtained (please see column 1 of Table 1). For genes related to a certain disease class, those without Ensembl IDs occurring in the PPI network reported in STRING were discarded. Column 2 of Table 1 lists the number of remaining genes related to each one of the 22 disease classes. The detailed codes of the related genes are provided in Supplementary Material I (in Supplementary Material available online at http://dx.doi.org/10.1155/2016/4895476).

### 2.2. Genes Modulated by PM2.5

Genes that can be modulated by PM2.5 were retrieved from Gualtieri et al.'s study [6], in which 177 differentially expressed genes were reported to be modulated by PM2.5 winter and summer with *p* value < 0.05 and 43 differentially expressed genes were reported to be modulated by PM2.5 with log 2 fold change <−0.5 or >0.5. We combined these differentially expressed genes and obtained 189 genes that are provided in Supplementary Material II. For convenience, they were called PM2.5 genes in this study and comprised the gene set *D*_PM_.

### 2.3. PPI

PPI information is a widely used type of information to investigate various protein-related and gene-related problems. Many investigators have built several effective computational methods to deal with different biological problems, such as protein functions prediction [8–12] and disease gene identification [13–19]. Most of the methods were based on a widely accepted fact that two proteins in a PPI always share similar functions [8, 9, 11, 19]. To uncover the associations between 22 disease classes and PM2.5, it is natural to analyze the relationships between their related genes, while the PPI information is one of the most suitable materials to address this problem.

In this study, we used the PPI information reported in STRING (version 9.0, http://string.embl.de/) [20], a large online database containing a large number of PPIs for several organisms. Comparing to the PPIs reported in some other databases, such as Database of Interaction Proteins (DIP) [21], BioGRID [22], which only provide PPIs validated by experiments, PPI used in this study can measure associations between proteins from the point of view of both the protein physical properties and the protein functional properties because they are derived from the following four sources: (1) genomic context, (2) high-throughput experiments, (3) (conserved) coexpression, and (4) previous knowledge. All PPI information was contained in a file called “protein.links.v9.0.txt.gz” which can be downloaded from STRING. Each PPI consists of two proteins, represented by Ensembl IDs, and one score with range between 150 and 999, indicating the strength of the interaction. For proteins *p*_1_ and *p*_2_, let us denote the score of the PPI between them by *I*(*p*_1_, *p*_2_). If *p*_1_ and *p*_2_ are identical, *I*(*p*_1_, *p*_2_) was set to 1,000, while it was set to zero if the interaction between them is not reported in STRING.

### 2.4. Measuring the Associations between Disease Genes and PM2.5

To analyze the associations between each OMIM disease class and PM2.5, we can investigate the associations between genes related to each disease and PM2.5 genes, thereby inducing the likelihood of genes modulated by PM2.5.

For a gene *g* related to one OMIM disease class, if it has strong associations with one PM2.5 gene, it may have similarity functions with the PM2.5 gene, suggesting that it may be modulated by PM2.5. Thus, we calculated the maximum interaction score for each disease gene *g* as follows:

(1) $$\begin{array}{c} Q\left(\;g\;\right)=\max\left\{\;I\left(\;g,g^{\prime }\;\right):g^{\prime }\in D_{PM}\;\right\}. \end{array}$$

Obviously, this score measures the associations between *g* and PM2.5 genes. A high score means that there is at least one PM2.5 gene that is highly related to *g*. Because each PM2.5 gene can be modulated by PM2.5, it can infer that *g* can be modulated by PM2.5 with high probability.

Each disease gene measured the associations between it and PM2.5 by investigating the PPIs between it and PM2.5 genes. However, some disease genes may occupy the special locations in the PPI network, meaning that they are highly related to any gene. In this case, the maximum interaction score calculated by (1) cannot reflect the reality. In this case, another measurement should be employed for each disease gene *g*. To obtain this measurement, we randomly produced 1,000 sets of genes, denoted by *D*_1_, *D*_2_,…, *D*_1000_, and each of them had the same size of *D*_PM_. For gene set *D*_*i*_, calculate the score as follows:

(2) $$\begin{array}{c} Q_{i}\left(\;g\;\right)=\max\left\{\;I\left(\;g,g^{\prime }\;\right):g^{\prime }\in D_{i}\;\right\}. \end{array}$$

Accordingly, there is one score for *D*_PM_ and 1,000 scores for 1,000 randomly produced gene sets. The measurement, namely, permutation FDR (False Discovery Rate), was defined to be the proportion of 1,000 scores for randomly produced gene sets that were larger than the score for *D*_PM_. For convenience, this measurement was denoted by FDR(*g*) for disease gene *g*. Obviously, small permutation FDR for a gene suggests that its position in the whole PPI network is not special and it is highly related to PM2.5 if its maximum interaction score is high.

### 2.5. Measuring the Associations between Diseases and PM2.5

For each OMIM disease class, there are several genes related to it. Each gene has measured its associations with PM2.5 by calculating the maximum interaction score and permutation FDR. If a gene received a small permutation FDR, it may be highly related to PM2.5. In view of the fact that 0.05 is always selected as the cutoff value for the significance level in the traditional test and has been applied in some studies [14, 15, 23], we also set 0.05 as the threshold for the permutation FDR to filter most related disease genes among the genes related to an OMIM disease class. Because the numbers of genes related to 12 disease classes are of great difference, it is not reasonable to measure the associations between diseases and PM2.5 only considering the number of selected genes. The proportion of the selected genes to all genes is more suitable. It is clear that if a disease received a high proportion, that is, many genes related to this disease are closely related to PM2.5, it may have strong associations with PM2.5 and PM2.5 can be a potential etiologic factor of this disease.

## 3. Results and Discussion

**Figure 1:**
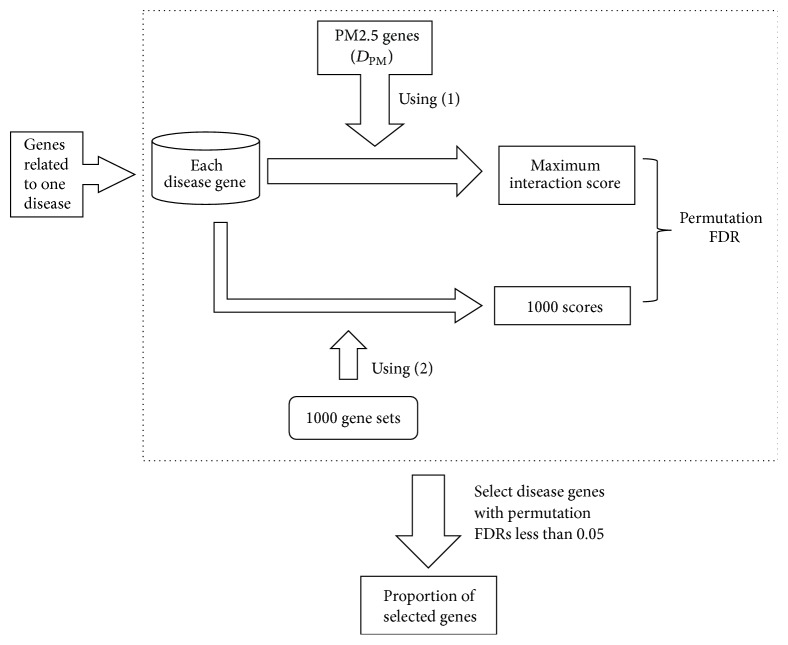
A flowchart to illustrate the procedures of our method.

In this study, we proposed a computational method to measure the associations between 22 disease classes and PM2.5. The whole procedures are illustrated in Figure 1. This section would give the detailed results and the discussion based on them.

### 3.1. Results of the Associations between Disease Genes and PM2.5

As described in Section 2.4, we calculated two measurements: maximum interaction score and permutation FDR for each disease gene, to quantify the associations between it and PM2.5. These values are listed in Supplementary Material III.

### 3.2. Results of the Associations between Diseases and PM2.5

**Figure 2:**
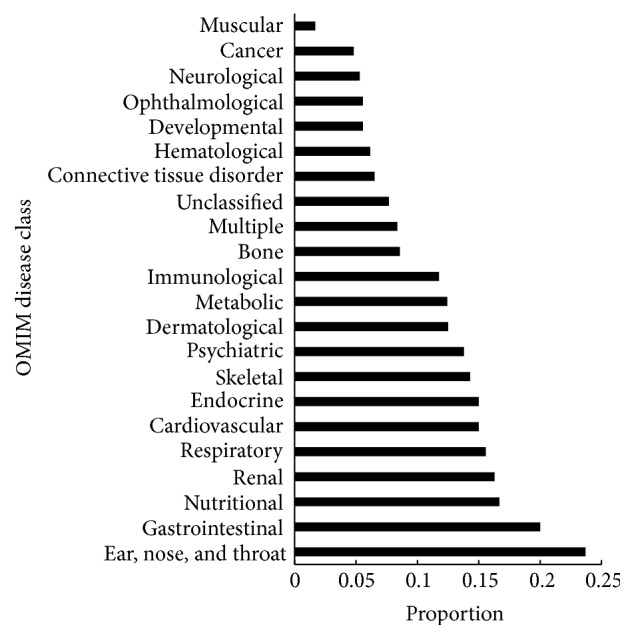
Bar chart illustrating the proportion of the selected genes to all disease genes for each disease class.

For genes related to one disease class, we excluded those with permutation FDR larger than or equal to 0.05. The proportion of the remaining genes was calculated to measure the associations between the disease and PM2.5. These proportions are listed in Table 2. For easy observation, a bar chart (Figure 2) was also plotted to illustrate the proportions for all 22 disease classes. It can be seen that the disease class “ear, nose, and throat” is the leader disease which is deemed to be closely related to PM2.5, followed by disease classes “gastrointestinal,” “nutritional,” and so forth. The following selection would give the detailed discussion on these findings, suggesting that our method can recover some known results and also imply some new results.

### 3.3. Analysis of the Results

According to the results listed in Table 2, “ear, nose, and throat,” “gastrointestinal,” “nutritional,” “renal,” “respiratory,” and “cardiovascular” classes are the top six disease classes that are most related to PM2.5. On the other hand, “developmental,” “ophthalmological,” “neurological,” “cancer,” and “muscular” classes are the least five disease classes. This section would give some evidences to show that some top disease classes are closely related to PM2.5.

#### 3.3.1. Nasal Biopsies Exposed to PM2.5 Show Squamous Metaplasia and Some Biochemical Changes (e.g., the Increasing Secretion of Amphiregulin and IL-8)

Nose and throat are directly exposed to outside pollutants. Also, running nose, blocked nose, and cough are often reported in patients living in polluting areas. For example, an experiment based on nasal biopsies of children from polluted areas in Southwest Metropolitan Mexico City has revealed that their biopsies displayed squamous metaplasia and decreased numbers of goblet and ciliated cells, and so on [24]. Another experiment using apical membranes of well-differentiated human nasal epithelial (HNE) cells exposed to PM2.5 suggested that PM2.5 can stimulate both amphiregulin and IL-8 secretion [25].

#### 3.3.2. Air Pollutants Are Associated with Intestinal Diseases, Such As Appendicitis and Children Acute Diarrheal Disease

The influence of PM2.5 on the intestinal system is poorly investigated until now. Despite the fact that the oral route can be accessible easily, large fractions of air pollutants will also enter and further impact the intestine, because of systemic inflammation [26]. Fortunately, there are still studies that show an association between air pollutants and different aspects of intestinal diseases. Kaplan and his colleagues showed that the incidence of appendicitis is related to some pollutants, such as SO_2_ and NO_2_ in summer [27]. Orazzo and his colleagues reported that children acute diarrheal disease is positively corrected with SO_2_ and CO [28].

#### 3.3.3. Exposure to PM2.5 Increases Risk to Diabetic and Obese Individuals

Nutritional diseases refer to any of the nutrient-related diseases and conditions that cause illness in humans, including eating disorders and obesity, deficiencies or excesses in the diet, and chronic diseases such as cardiovascular disease, diabetes mellitus, and hypertension. Schneider et al. claimed that exposure to PM2.5 is detrimental to diabetic individuals, in detail, patients with type 2 diabetes shown endothelial cell dysfunction after exposure to PM2.5 [29]. Also, experimental data suggest that the bad effects of inhaled PM2.5 can be exacerbated by a high-fat diet [30], and increased risk is accompanying obese people [31].

#### 3.3.4. Heavy Metals in PM2.5 (e.g., Lead and TiO_2_) Can Damage Kidneys

PM2.5 contains many heavy metals that can damage kidneys. Even though lead concentration is dependent on the sampling location, lead often exists in PM2.5. Report has shown that exposure to lead can cause kidney damage [32]. Also, titanium dioxide (TiO_2_) is frequently used in a wide range of plastics, paints, and paper. It is shown that it can induce serious swelling in rats' renal glomerulus and lead to significant lesions of kidneys [33].

#### 3.3.5. PM2.5 Increases Risk of Cardiovascular Diseases Supported by Disease Statistics and Associations in Genes (e.g., Cardiovascular Relevant Gene DSP Is Related to PM2.5 Gene JUP)

Sufficient evidence has proved that PM2.5 is associated with cardiovascular diseases. A study in the northeastern United States confirmed that risks in all-cause coronary heart disease (CHD) are increasing when the exposure to PM2.5 is increasing [34]. In Massachusetts, an association between occurrence of acute myocardial infarction (AMI, one of the specific cardiovascular diseases) and long-term exposure to area PM2.5 has been proven [35]. In this study, cardiovascular diseases are also predicted to be highly related to PM2.5.  Table 3 lists the maximum interaction scores and permutation FDRs of selected genes related to cardiovascular diseases, from which we can see that the total of 18 genes related to cardiovascular diseases were identified to be closely related to PM2.5 by our method. Here, some genes that are most relevant to PM2.5 would be discussed. For example, gene DSP (desmoplakin, a component of functional desmosomes) plays a crucial role in intercellular junctions between adjacent cells. DSP is significantly related to PM2.5 gene JUP. Research has found that mutations in DSP can cause arrhythmogenic right ventricular dysplasia/cardiomyopathy (ARVD/C) and increase risk of sudden death. More importantly, mutations of JUP itself have also been identified in patients with ARVD/C [36], which further explains that PM2.5 might increase risks of cardiovascular diseases. Another example is PTGS2. The abnormal expression of PTGS2 is found when cells are exposed to PM2.5, and the PM2.5 gene PTGS2 is related to PTGIS (a member of the cytochrome P450 superfamily of enzymes), which encodes monooxygenases that are involved in synthesis of lipids, such as cholesterol and steroids. Patients who are at high risks of developing stroke and myocardial infarction often have mutations in PTGS2 or abnormal expression of PTGIS [37].

Here, two considerations relevant to our research should be mentioned. First, we should confirm that current findings that PM2.5 can aggravate lung and heart diseases, such as respiratory symptoms and heart attacks, are easily influenced by subjective judgments and unknown factors. This is partly because most findings between diseases and PM2.5 are based on hospital admissions and information from questionnaires, which could be largely influenced by weather, epidemic diseases, and other factors. In addition, few researches have focused on other diseases, such as gastrointestinal diseases, due to no enough resources and reasonable methods for them to dig into all these diseases. Thus, it will be unwise to conclude which disease is mostly influenced by PM2.5, without ruling out other diseases' associations with PM2.5 due to lack of relevant research. Second, we adopted 22 disease classes based on Maurizio Gualtieri and his colleges' study. The mixtures of several diseases to one disorder might cover some relevant information or add some irrelevant information. Despite the existence of the flaw, our goal is to research associations between PM2.5 and most human diseases that have not been executed before. Since no other solution has been found to solve this problem well, we assume that this method is accepted, relevant, and accurate.

## 4. Conclusions

This contribution gives an intensive investigation on the linkages between PM2.5 and several diseases. For each disease, its association with PM2.5 was measured by its related genes and PM2.5 genes using the protein-protein interaction information. Our method affirms that some diseases are highly related to PM2.5. It is hopeful that the new findings in this study may give new ways to study the causal relationship between diseases and PM2.5. In addition, our method used the PPIs to evaluate the associations between PM2.5 and several diseases. In future, we will focus on integrating some advanced network algorithms, such as the shortest path algorithm and random walk algorithm, into the PPI network to build more effective methods for addressing the problem.

## Supplementary Material

The Supplementary Material consists of three files. In detail, Supplementary Material I lists genes related to 22 OMIM disease classes; Supplementary Material II lists 189 genes modulated by PM2.5; Supplementary Material III lists maximum interaction score and permutation FDR for each disease gene.

## Figures and Tables

**Table 1:** The number of genes related to each of OMIM classes of disease.

OMIM disease class	Number of related genes
Bone	35
Cancer	166
Cardiovascular	120
Connective tissue disorder	46
Dermatological	72
Developmental	54
Ear, nose, and throat	38
Endocrine	80
Gastrointestinal	25
Hematological	65
Immunological	85
Metabolic	177
Multiple	179
Muscular	59
Neurological	226
Nutritional	18
Ophthalmological	90
Psychiatric	29
Renal	43
Respiratory	45
Skeletal	56
Unclassified	13

**Table 2:** The associations between the 22 disease classes and PM2.5.

OMIM disease class	Proportion
Ear, nose, and throat	0.2368
Gastrointestinal	0.2000
Nutritional	0.1667
Renal	0.1628
Respiratory	0.1556
Cardiovascular	0.1500
Endocrine	0.1500
Skeletal	0.1429
Psychiatric	0.1379
Dermatological	0.1250
Metabolic	0.1243
Immunological	0.1176
Bone	0.0857
Multiple	0.0838
Unclassified	0.0769
Connective tissue disorder	0.0652
Hematological	0.0615
Developmental	0.0556
Ophthalmological	0.0556
Neurological	0.0531
Cancer	0.0482
Muscular	0.0169

**Table 3:** Detailed information on 18 cardiovascular related genes with permutation FDR less than 0.05.

Disease gene	Maximum interaction score	Permutation FDR	Most related PM2.5 gene
DSP	999	0.009	JUP
PTGIS	977	0.011	PTGS2
EPHX1	995	0.012	CYP1A1
PPARG	999	0.017	PPARGC1A
OLR1	944	0.018	CCL2
PKP2	984	0.024	JUP
TCF4	994	0.028	ID1
NR3C2	953	0.029	SGK1
TCF7L2	997	0.029	JUP
ELN	993	0.033	FBN2
NEUROD1	939	0.035	ID2
F5	998	0.036	PROC
F13A1	970	0.037	FGG
BMPR2	999	0.039	BMP6
PNMT	952	0.04	EGR1
IL6	999	0.042	IL6ST
TNFSF4	859	0.042	STAT4
HNF1B	947	0.044	SOX9
