# Supplementary material for: The Use of Protein-Protein Interactions for the Analysis of the Associations between PM2.5 and Some Diseases
Source: Biomed Res Int. 2016 May 8;2016:4895476. doi: 10.1155/2016/4895476 (PMC4875974; doi:10.1155/2016/4895476)
Supplement: Supplementary file 1 — The Supplementary Material consists of three files. In detail, Supplementary Material I lists genes related to 22 OMIM disease classes; Supplementary Material II lists 189 genes modulated by PM2.5; Supplementary Material III lists maximum interaction score and permutation FDR for each disease gene. [file 4895476.f1.zip › Supp-I.pdf]

Supplementary Material I. Genes related to 22 OMIM disease classes

1. Bone

COMP

COL1A1

VDR

FGF23

TYROBP

TCIRG1

TNFRSF11A

ANKH

SLC26A2

LRP5

COL1A2

TNFRSF11B

EIF2AK3

LEMD3

ANO5

KLK4

CYP2R1

COL9A3

EXT2

CALCR

WISP3

GNAS

COL9A2

TREM2

COL11A2

ALPL

EXT1

KL

COL2A1

AMELX

SQSTM1

GALNT3

ENAM

DSPP

MATN3

2. Cancer

NME1

RB1CC1

GOLGA5

ESR1

AURKA

HNF1B

CD82

CCND1

MLH1

MSH2

ODC1

MSH6

FLT3

IRF1

PTPN12

PDGFRL

TSG101

KRAS

VHL

PDGFRA

APC

HNF1A

TMEM127

BRIP1

IL1RN

TLR2

FGFR3

BARD1

IDH1

DIRC2

PALB2

CDH1

PDGFRB

FLT4

MSR1

AXIN1

MAP3K8

CCDC6

EP300

IL1B

CHIC2

KDR

KIF1B

PIK3CA

LZTS1

PMS2

ETV6

RB1

ZFHX3

TP53

ERBB2

SH3GL1

AKT1

PRCC

RASA1

ARHGAP26

EGFR

DLC1

ATM

FEZ1

FLCN

CASP10

BACH1

BUB1B

KIT

BRAF

BAX

CDS1

NPM1

RUNX1

PHB

CYP2A6

ANTXR1

ASPSCR1

AXIN2

BCR

RNF139

MN1

OGG1

PPM1D

DLEC1

TERT

HRAS

PPP2R1B

WHSC1L1

GDNF

MET

LPP

SMARCE1

STK11

PLAG1

TACC3

PCM1

PDGFB

MXI1

WT1

PTCH1

RAD54B

HIP1

NQO2

TRIM24

PTPN11

SMAD4

RNF6

XRCC3

ATR

CTNNB1

NF2

GATA2

BLM

RET

MLH3

ERCC6

MLF1

LIG4

IGF2R

RASSF1

BRCA1

NF1

TRIM33

CASP8

PRKAR1A

NTRK1

MAX

TGFBR2

NUP214

CDKN2A

FH

PARK2

RNASEL

SDHC

NRAS

SUFU

GPC3

SH2D1A

PTEN

RAD54L

MINPP1

PTCH2

COL4A6

TLR4

AR

PLA2G2A

PAX7

SDHB

SDHD

GATA1

MYC

EXT1

BRCA2

IRF4

JAK2

CHEK2

PAX3

HMMR

ARHGEF12

MAD1L1

WWOX

MCC

DCC

NCOA4

PTPRJ

MUTYH

CBFB

GMPS

KLF6

3. Cardiovascular

ABCA1

ABCC8

ABCC9

ADD1

ADRB1

AGT

AGTR1

AKT2

ALOX5AP

ANK2

APOA2

ATP1B1

BMPR2

CAV3

CDKAL1

CFC1

CSRP3

CYP3A5

DES

DMD

DSP

DTNA

ECE1

ELN

ENG

ENPP1

EPHX1

EPHX2

ESR1

EYA4

F13A1

F2

F5

F7

FH

GATA4

GATA6

GCGR

GCK

GCLC

GCLM

GDF1

GHR

GIP

GJA1

GNAI2

GNB3

GPD2

HGF

HMGA1

HNF1A

HNF1B

HNF4A

IGF2BP2

IL6

IRS1

IRS2

ITIH4

JAG1

KCNJ11

KCNJ2

KCNMB1

KCNQ1

KRIT1

LDLR

LGALS2

LIPC

LIPH

LMNA

LRP8

LTA

MAPK8IP1

MEF2A

MTNR1B

MYBPC3

MYH7

MYL2

MYL3

MYLK2

NAT1

NET1

NEUROD1

NKX2-5

NKX2-6

NOS2

NOS3

NOTCH3

NR3C2

OLR1

PAX4

PDE4D

PKP2

PNMT

PPARG

PRKAG2

PRKAR1A

PRKCH

PSMA6

PTGIS

PTPN1

RASA1

RETN

RGS5

RYR2

SCN5A

SCO2

SELE

SGCD

SLC2A4

SLC6A2

TBX1

TCAP

TCF4

TCF7L2

TEK

TNFSF4

TNNI3

TPM1

WFS1

ZFPM2

4. Connective tissue disorder

ABCC6

ADAMTS10

ADAMTS2

ANKH

ANTXR2

ARSE

ASPN

B4GALT7

CD244

COL11A2

COL1A1

COL1A2

COL3A1

COL5A1

COL5A2

COL6A1

EBP

ELN

ENPP1

FBLN5

FBN1

FGFR2

FLNB

FRZB

GNPAT

HGF

HLA-B

HLA-DRB1

IL10

IL6

KRT13

KRT4

MIF

NFKBIL1

PADI4

PLOD1

PTPN22

SCT

SLC22A4

SLC26A2

SMARCAL1

SOS1

TGFBR1

TNXB

XYLT1

XYLT2

5. Dermatological

ABCA12

ADAR

ALOX12B

ATP2C1

CDH3

CDSN

COL17A1

COL7A1

CYLD

DDB2

DKC1

DSG1

DSG4

DSP

EDA

EDAR

ERCC2

ERCC3

ERCC4

ERCC5

FLCN

GJB2

GJB3

GJB4

GJB6

HLA-A

HLA-B

HLA-C

HR

IKBKG

ITGA6

ITGB4

KRT1

KRT10

KRT14

KRT16

KRT17

KRT2

KRT5

KRT6A

KRT6B

KRT81

KRT83

KRT86

KRT9

LAMA3

LAMB3

LAMC2

LMNA

LTA

MBTPS2

MLPH

MMP1

MPLKIP

MYO5A

NOD2

PKP1

PLEC

POLH

PSTPIP1

PVRL1

RAB27A

SAT1

SLC39A4

SLURP1

SOX18

SPINK5

TGM1

TMC6

TMC8

TYR

ZMPSTE24

6. Developmental

MIF

AMH

CCL2

MYH3

TWIST1

MKKS

GDF1

TNNI2

AMHR2

CFC1

CYP1B1

SIX3

MTRR

GATA6

TNNT3

MED13L

NIPBL

NODAL

LHCGR

T

SHH

TSC1

ZEB2

IGF1

INSR

PITX2

ESCO2

LEMD3

TBX5

VANGL1

FUZ

EZH2

NKX2-5

TBX1

PTPN11

ATR

TPM2

MTR

IRF6

NSDHL

TBX22

HSD17B3

ZIC2

MTHFR

PAX6

CDKL5

MSX1

TRPS1

UBE3A

MECP2

NKX2-6

EZH1

TCOF1

FGFR2

7. Ear Nose Throat

USH1C

MYO15A

MYH9

COCH

WFS1

CTSC

POU4F3

GJB6

GRHL2

KCNQ4

TECTA

SLC26A4

MYO3A

OTOF

TMPRSS3

TMC1

MYO1A

FOXI1

ATP2B2

TMIE

ACTG1

EYA4

DFNB31

PCDH15

KCNJ10

MYO6

POU3F4

GJB3

COL11A2

ESPN

GJB2

OTOA

DIAPH1

CDH23

DSPP

MYO7A

STRC

MYH14

8. Endocrine

AQP2

TG

RETN

SLC5A5

HGF

GCK

HNF1B

GNRHR

WFS1

SCNN1A

MAPK8IP1

BMP15

FSHB

MTNR1B

HNF1A

IL6

SYCP3

DHH

CDKAL1

FGD2

STAR

AIP

PTH

PPP1R3A

PPARG

CYP11B1

STAT5B

SSTR5

NEUROD1

HESX1

CASR

LIPH

TSHR

LIPC

SCNN1G

CTLA4

IRS1

MRAP

HMGA1

GPD2

PROP1

HNF4A

SUMO4

SLC2A4

TPO

MC2R

AVPR2

PAX4

KCNJ11

SCNN1B

TCF4

THRB

NR3C2

PTPN22

ENPP1

CACNA1S

TBCE

TBX19

TCF7L2

TTF2

CYP17A1

GNAS

STX16

PTPN1

ITPR3

AR

FOXE1

IRS2

FOXP3

PTF1A

GATA3

AVP

IGF2BP2

ABCC8

CAPN10

AKT2

GCGR

FSHR

OAS1

PAX8

9. Gastrointestinal

CFTR

HGF

PRKCSH

IL6

BAAT

ABCB11

FGF10

ABCB4

EPHX1

ATP8B1

KRT8

SPINK1

HSD3B7

NOD2

UGT1A1

PRSS1

CIRH1A

VPS33B

EDNRB

SLC26A3

RET

SEC63

CTRC

TJP2

KRT18

10. Hematological

THPO

UNC13D

MYH9

GSS

F9

RPS19

EPOR

EPX

IFNG

GCLC

GGCX

SLC19A2

NT5C3A

A4GALT

LMAN1

APOE

ABCB7

VHL

GIF

PDGFRA

CYP2C9

FLT4

ITGA2B

SLC4A1

ELANE

CP

TF

GCNT2

LBR

AQP3

AMN

RUNX1

CYP2A6

ACHE

GP9

FGB

FGA

CD36

RPS14

AQP1

CFHR1

FOXC2

GP1BA

TPO

BSG

HBB

SH2B3

CDAN1

CR1

CFHR3

CFH

CPN1

RHAG

ADAMTS13

MPL

FHL3

ATRX

MASTL

ANKRD26

GATA1

WAS

CUBN

JAK2

VKORC1

GP1BB

11. Immunological

ADA

AICDA

AIRE

ATM

BANK1

BTK

C4A

CASP10

CASP8

CCL11

CCL2

CCL3

CD209

CD40

CD40LG

CD8A

CEBPE

CISH

CX3CR1

CXCL12

CXCR1

CXCR4

CYBA

CYBB

DCLRE1C

DNASE1

FAS

FCGR2B

HAVCR1

HGF

HLA-C

HLA-DRB1

ICOS

IFNG

IFNGR1

IFNGR2

IL10

IL12B

IL12RB1

IL13

IL21R

IL4R

IL6

ITGB2

JAK3

KIR3DL1

KIR3DS1

LIG4

MC3R

MEFV

MPO

MRE11A

MS4A2

MVK

NCF1

NCF2

NLRP3

PARK2

PHF11

PLA2G7

PTPN22

PTPRZ1

RAC2

RAG1

RAG2

RFX5

RFXANK

RFXAP

SELP

SERPING1

SLC11A1

SP110

SPINK5

STAT1

TAP1

TAP2

TAPBP

TIRAP

TLR2

TLR3

TLR5

TNFRSF13B

TNFRSF1A

UNG

WAS

12. Metabolic

AASS

ABCA1

ABCC2

ABCC8

ABCD1

ABCG5

ABCG8

ABHD5

ACADM

ACADS

ACADVL

ACAT1

ADSL

AGPAT2

AGXT

ALDH3A2

ALDH4A1

ALG1

ALG12

ALG2

ALG3

ALG6

ALG8

ALPL

AMN

AMT

APOA1

APOA2

APOA5

APOB

ARG1

ASL

ASPA

ASS1

ATIC

ATP7B

AUH

B4GALT1

BCKDHA

BCKDHB

BCS1L

BSCL2

BTD

CAV3

CETP

COG7

CP

CPS1

CPT1A

CPT2

CTH

CYP11B2

CYP27A1

D2HGDH

DBH

DBT

DDC

DHCR24

DMGDH

DPAGT1

DPM1

ECM1

EPHX2

ETFA

ETFB

ETFDH

ETHE1

FH

FMO3

FTCD

GAA

GALK1

GALT

GBA

GBE1

GCDH

GCK

GCSH

GHR

GK

GLDC

GLUD1

GNE

GNMT

GNPTAB

GNS

GRHPR

GSS

GYS2

H6PD

HAL

HEXA

HEXB

HFE

HGD

HLCS

HMGCS2

HPRT1

HSD17B10

HSD17B4

HYAL1

IDUA

ITIH4

IVD

L2HGDH

LAMP2

LCAT

LDLR

LDLRAP1

LIPI

LMNA

MADD

MAN2B1

MANBA

MCCC1

MCCC2

MCM6

MCOLN1

MGAT2

MLYCD

MMAA

MMAB

MOCS1

MPDU1

MPI

MTHFR

MTR

MTRR

MUT

NAGA

NAGS

NEU1

NPC1

NPC2

OCRL

OPA3

OTC

PANK2

PC

PCCA

PCCB

PCSK9

PDX1

PHEX

PHGDH

PHKB

PMM2

PPARG

PPOX

PPP1R3A

PRODH

PYGM

RP1

RPIA

SCD

SGSH

SLC17A5

SLC22A5

SLC25A13

SLC25A15

SLC2A1

SLC2A2

SLC35C1

SLC40A1

SLC5A1

SLC6A19

SLC7A7

SMPD1

SUMF1

SUOX

TALDO1

TFR2

UGT1A1

UROS

USF1

WFS1

XDH

13. Multiple

USH1C

HOXA11

SNAI2

MYOC

KCNQ1

AAAS

MYH9

SALL4

HOXA13

WNT3

CTSC

MGP

NDUFS1

NDUFB3

BBS9

DNAI1

EGR2

BBS2

MKKS

SBDS

PEX1

SALL1

TYMP

GFAP

JAG1

AP3B1

SDS

TBX3

BRIP1

CFC1

NDUFAF1

TRIM37

CREBBP

TYR

LIFR

FOXRED1

NDUFS3

DGUOK

RAB3GAP1

AGPS

BBS7

TP63

FRAS1

SDHA

ERCC8

CUL7

CACNA1C

BBS4

DYM

NDUFS6

RASA1

NDUFB9

FREM2

MYCN

NUBPL

BACH1

ZIC3

BBS5

CLDN1

MITF

HESX1

NDUFAF2

NDUFS4

WRN

TSC1

TAZ

NOD2

SEMA3E

INSR

PITX2

UGT1A1

USH2A

CEP290

LEMD3

PMP22

HRAS

JUP

NDUFA11

MID1

PEX7

KCNJ1

BBS12

USH1G

CLRN1

NDUFV1

RAI1

NDUFAF3

LAMA3

PRX

HPS1

FANCB

NDUFV2

NOG

DNMT3B

PHF6

WT1

TBX1

PTCH1

NDN

FOXL2

EDNRB

NLRP3

ARL6

PTPN11

ATP7A

LMX1B

DHCR7

ERCC6

LIG4

CHM

COL11A1

VPS13B

PRKAR1A

BCS1L

COL18A1

MPZ

KIAA1279

PCDH15

PAX2

TBCE

IRF6

AHI1

NDUFS2

LOR

NDUFAF4

FLNA

L1CAM

PDZD7

GPC3

GNAS

BSND

NDUFA1

INPP5E

PTEN

POMGNT1

POMT1

ZMPSTE24

ATRX

CCDC28B

TRIM32

HSPG2

COL11A2

CLCNKB

ROR2

FOXP3

PUS1

GATA1

NDUFAF5

BCOR

SKI

SDHAF1

ARX

RPS6KA3

DSP

BRCA2

COL2A1

TTC8

SLC12A1

GJB2

FANCA

LYST

PAX3

CRLF1

MKS1

BBS10

TFAP2B

KIF7

FGFR1

GRIP1

CDH23

GPR98

SMS

LCA10

TMEM67

CHD7

FGFR2

CDKN1C

ATPAF2

GFM1

14. Muscular

PABPN1

SGCG

SCG3

FKTN

CRYAB

MYOT

MYH2

IGHMBP2

CHRND

CHRNA1

SGCA

DGUOK

TNNT1

CHRNE

COL6A3

MTMR14

RAPSN

COL6A2

CHRNB1

SMAD1

TCAP

PLEC

FKRP

CHAT

SGCD

CLCN1

CAV3

DMPK

LARGE

MYH7

ATP2A1

VCP

RYR1

DNM2

BSCL2

DMD

SEPN1

COL6A1

ACTA1

LMNA

EMD

MTM1

CPT2

POMT1

DES

TRIM32

MUSK

AR

SMN1

SMN2

SGCB

GARS

GNE

CAPN3

NEB

DYSF

SCN4A

LAMA2

VAPB

15. Neurological

ABCD1

ACE

ACSL4

ALMS1

ALS2

AMN

APBB2

APOA1

APOE

APP

APTX

ARFGEF2

ARHGEF10

ARHGEF6

ARSA

ARX

ASPM

ASPN

ATCAY

ATL1

ATN1

ATP1A2

ATP1A3

ATP7A

ATXN1

ATXN2

ATXN3

ATXN7

BCS1L

BLMH

BSCL2

CACNA1A

CACNA1S

CCM2

CDK5RAP2

CENPJ

CHM

CHRNA4

CHRNB2

CILP

CLCN2

CLN3

CLN5

CLN6

CLN8

COL11A1

COL4A1

COL9A2

COL9A3

COLQ

COX15

CP

CRBN

CST3

CSTB

DCTN1

DCX

DNM2

DRD2

EDNRA

EFHC1

EGR2

EIF2B1

EIF2B2

EIF2B3

EIF2B4

EIF2B5

EPM2A

ESR1

EYA1

FGA

FGF14

FLNA

FOXP2

FOXRED1

FTL

FTSJ1

FXN

GABRG2

GALC

GAN

GARS

GBA

GCH1

GDAP1

GJB1

GJC2

GLRA1

GPR56

GPR98

GSN

HCFC1

HFE

HLA-DQB1

HLA-DRB1

HOXA1

HSPB1

HSPB8

HSPD1

HSPG2

IGBP1

IKBKAP

ITM2B

JPH3

KCNMA1

KCNQ2

KCNQ3

KDM5C

KIF1B

KIF5A

KRIT1

L1CAM

LGI1

LITAF

LRPPRC

LRRK2

LYZ

MAPT

MCPH1

ME2

MECP2

MFN2

MLC1

MPO

MPZ

MR1

MTMR2

NDRG1

NDUFA10

NDUFA12

NDUFA2

NDUFA9

NDUFAF2

NDUFAF6

NDUFS3

NDUFS4

NDUFS7

NDUFS8

NEFH

NHLRC1

NIPA1

NKX2-1

NLGN3

NOS3

NTRK1

OPHN1

PAFAH1B1

PAK3

PANK2

PARK2

PARK7

PDC

PDCD1

PDHA1

PER2

PEX12

PEX5

PHYH

PINK1

PLAU

PLP1

PMP22

PNKD

POLG

PPP2R2B

PPT1

PQBP1

PRKCG

PRPH

PRSS12

PSEN1

PSEN2

RELN

RET

ROBO3

RYR1

SACS

SBF2

SCN1A

SCN1B

SCN2A

SCN4A

SCN9A

SDHA

SERPINI1

SETX

SGCE

SH3TC2

SHH

SIX3

SLC12A6

SLC16A2

SLC19A3

SLC25A19

SLC25A22

SLC6A8

SMAD1

SMS

SNAP29

SNCA

SNCB

SOD1

SORL1

SOX10

SOX3

SPAST

SPG20

SPG21

SPG7

SPTLC1

SURF1

SYN1

TBP

TDP1

THBS2

TIMM8A

TNF

TOR1A

TPP1

TTF1

TTPA

VAPB

VLDLR

VPS13A

WNK1

ZNF81

16. Nutritional

NR0B2

SIM1

UCP1

POMC

PPARG

AGRP

MC4R

ADRB2

PCSK1

PPARGC1B

UCP3

GHRL

ADRB3

SDC3

PYY

ENPP1

SLC6A14

HTR2A

17. Ophthamological

MYOC

GUCA1A

RP1

CRX

CRYBA1

TULP1

PRPH2

GJA8

GJA3

KRT12

APOE

GUCY2D

PRPH

RDH5

CYP1B1

RPE65

RGS9

OPTN

HSF4

CRYGD

PIKFYVE

VCAN

TIMP3

KERA

POLG

RLBP1

HMCN1

CNGA3

ROM1

CRYGC

CRYAA

LRP5

RHO

SLC45A2

RP9

PHOX2A

CTDP1

CA4

BEST1

PITX2

PRPF8

COL8A2

DRD5

LAMB2

PLG

CFHR1

CNGB3

OPA3

SOX2

PRPF31

FBN1

CHST6

RGS9BP

FOXE3

FSCN2

NYX

CERKL

FBLN5

IMPDH1

EFEMP1

CHM

OPA1

KIF21A

CRB1

CFHR3

PLEKHA1

AIM1

ELOVL4

OPN1MW

OPN1LW

PITX3

ABCA4

TACSTD2

CACNA1F

VSX1

FTL

NDP

RPGR

CYP4V2

PAX6

NHS

FOXC1

AIPL1

SPATA7

RPGRIP1

SAG

C1QTNF5

KRT3

TGFBI

PDE6B

18. Psychiatric

RTN4R

DRD4

DAO

DLX2

APOL2

CHI3L1

GABBR2

SLC6A4

AKT1

SLC6A3

HCRT

CYP2A6

DRD5

DYX1C1

TPH2

APOL4

DTNBP1

HTT

PRODH

COMT

DISC1

CHRNA4

NLGN3

DAOA

MTHFR

KIAA0319

HTR2A

DRD3

BDNF

19. Renal

SLC7A9

HNF1B

ACTN4

ATP6V0A4

SLC3A1

PKD1

INVS

SLC12A3

CLDN16

REN

AGTR1

ACE

SCNN1G

UMOD

IQCB1

NPHP1

DIAPH2

INSL3

CD151

SLC5A2

WT1

COL4A5

FOXL2

AVPR2

NPHP3

TRPC6

SCNN1B

SLC4A5

CD2AP

TRPM6

CACNA1S

AGT

NPHS2

PKHD1

CLCN5

SLC22A12

NPHP4

NPHS1

CTNS

COL4A3

COL4A4

ZNF365

SLC4A4

20. Respiratory

ADRB2

ALOX5

ASCL1

BDNF

CCL11

CCL2

CD209

CFTR

CISH

DNAH5

EDN3

FCGR2A

FLCN

GDNF

HLA-G

HMOX1

HNMT

IFNG

IFNGR1

IL12B

IL13

MC3R

MMP1

MUC5B

MUC7

NPSR1

PHF11

PHOX2B

PLA2G7

PTGDR

RET

SCGB1A1

SCGB3A2

SERPINA1

SFTPA1

SFTPA2

SFTPB

SLC11A1

SP110

TGFB1

TIRAP

TLR2

TNF

TSC1

TSC2

21. Skeletal

SCT

EFNB1

DLL3

TGFB1

COMP

GHR

MSX2

TBX4

GHSR

SOX9

HOXD10

MNX1

FGFR3

CILP

BMPR1B

TP63

EVC

DYM

CTSK

LBR

GJA1

SLC26A2

IHH

FLNB

SHH

SOST

LMBR1

NOG

FBLN1

SRA1

NPR2

COL9A3

EVC2

MESP2

OFD1

COL11A1

RUNX2

TRAPPC2

WISP3

PAX9

THBS2

IRF6

PRG4

FLNA

COL9A2

GDF5

ASPN

ROR2

BMP2

COL2A1

SHOX

MSX1

HOXD13

FGFR1

MATN3

FGFR2

22. Unclassified

CFTR

TAS2R16

RCBTB1

PLOD2

LRP5

ADH1B

SH3BP2

GABRA2

TSPYL1

PTEN

HTR2A

FSHR

FGFR2
