# Supplementary material for: The Use of Protein-Protein Interactions for the Analysis of the Associations between PM2.5 and Some Diseases
Source: Biomed Res Int. 2016 May 8;2016:4895476. doi: 10.1155/2016/4895476 (PMC4875974; doi:10.1155/2016/4895476)
Supplement: Supplementary file 1 — The Supplementary Material consists of three files. In detail, Supplementary Material I lists genes related to 22 OMIM disease classes; Supplementary Material II lists 189 genes modulated by PM2.5; Supplementary Material III lists maximum interaction score and permutation FDR for each disease gene. [file 4895476.f1.zip › Supp-II.pdf]

Supplementary Material II. 189 genes modulated by PM2.5

ABCA1

ACOT4

AFAP1

AGR3

AHR

AHRR

ALDH1A3

ALDH3A1

ANTXR1

AOF1

AQP3

ATP8B4

AVPI1

BASP1

BMP6

C13orf15

C14orf4

C21orf63

C5

C7orf46

CAST

CCDC68

CCL2

CCL5

CDH1

CDRT15L2

CP

CPNE4

CPS1

CSGALNACT1

CXCL5

CYP1A1

CYP1B1

DEFB1

DENND5B

DKK1

DNAJC12

DNAJC3

DUSP1

DUSP5

EDARADD

EFEMP1

EGR1

EPAS1

EREG

ETS2

FAM165B

FAM46A

FAT

FBN2

FGB

FGG

FGL1

FLJ22662

FLJ37644

FLJ42957

FOS

FOSL1

FRMD4A

FST

GAD1

GAGE1

GALNT4

GDF15

GJA1

GJB2

GLULD1

GPR175

GPR32

GPR37

GPR64

GPRC5A

GREM1

H19

HAS2

HIVEP2

HLA-DMA

HLA-DMB

HMOX1

ID1

ID2

ID3

ID4

IER3

IGFBP3

IL6ST

INSL4

ISG20

JAG1

JUN

JUP

KCNJ2

KCNK3

KCTD1

KDELC2

KRT4

LBH

LIF

LOC100129665

LOC100133332

LOC169834

LOC342897

LOC554207

LOC645638

LOC652968

LOC728431

LRP5

MAP7

MCCC1

MEGF6

MN1

MT1F

MT1X

MT2A

MUC5B

NAP5

NEDD9

NKD2

NPAS2

NPTX1

NPY1R

NR4A1

NR4A2

NTRK3

NTS

OAS1

PADI3

PDE3A

PEG10

PER2

PLA2G10

PLA2G4A

PLAC8

PMEPA1

PPARGC1A

PRICKLE2

PROC

PTGER2

PTGER4

PTGS2

PTP4A1

RAP1GAP

RFTN1

RGS17

RHOBTB1

RHOV

RNF128

S100P

SCARA5

SDPR

SERPINI1

SGK1

SHISA2

SIM2

SIPA1L2

SLC10A4

SLC12A2

SLC22A3

SLC2A1

SLC45A4

SLC7A2

SLC7A7

SNAP25

SNF1LK

SOX9

ST6GAL2

STAT1

STAT4

STK32B

SUSD2

SYT1

TAF13

TGFB1I1

TIPARP

TLE4

TLR3

TM4SF20

TMEM156

TSPAN8

TTC39A

TUBB2B

UBASH3B

UGCG

VGLL3

VIPR1

WISP2

XBP1

ZNF836

ZSWIM6
