# Supplementary material for: The Use of Protein-Protein Interactions for the Analysis of the Associations between PM2.5 and Some Diseases
Source: Biomed Res Int. 2016 May 8;2016:4895476. doi: 10.1155/2016/4895476 (PMC4875974; doi:10.1155/2016/4895476)
Supplement: Supplementary file 1 — The Supplementary Material consists of three files. In detail, Supplementary Material I lists genes related to 22 OMIM disease classes; Supplementary Material II lists 189 genes modulated by PM2.5; Supplementary Material III lists maximum interaction score and permutation FDR for each disease gene. [file 4895476.f1.zip › Supp-III.pdf]

Supplementary Material III. Maximum interaction score and permutation FDR for each disease gene

1. Genes related to bone

Gene	Maximum interaction score	Permutation FDR
LRP5	998	0.024
COL2A1	990	0.028
GNAS	966	0.054
COMP	952	0.04
COL1A2	930	0.316
COL1A1	927	0.322
CALCR	908	0.193
VDR	904	0.301
EIF2AK3	897	0.071
TNFRSF11B	834	0.086
TNFRSF11A	804	0.241
ALPL	779	0.182
COL9A2	696	0.084
ANKH	690	0.129
DSPP	673	0.275
TREM2	621	0.113

SQSTM1	619	0.451
FGF23	601	0.35
COL9A3	541	0.143
TCIRG1	541	0.348
WISP3	488	0.136
KL	488	0.46
AMELX	458	0.398
TYROBP	437	0.735
MATN3	408	0.31
EXT1	404	0.458
SLC26A2	353	0.368
LEMD3	330	0.528
COL11A2	294	0.063
EXT2	266	0.666
CYP2R1	262	0.362
GALNT3	246	0.43
KLK4	154	0.732
ANO5	0	1
ENAM	0	1

2. Genes related to cancer

Gene	Maximum interaction score	Permutation FDR
IRF1	999	0.005
CDH1	999	0.047
AXIN1	999	0.051
EP300	999	0.125
EGFR	999	0.14
PTPN11	999	0.098
CTNNB1	999	0.2
AR	999	0.073
JAK2	999	0.116
ESR1	998	0.138
VHL	998	0.052
IL1B	997	0.077
AKT1	997	0.221
SMAD4	997	0.068
TP53	995	0.6
MYC	994	0.295
CCND1	992	0.161
HRAS	992	0.125
RB1	991	0.232

PLA2G2A	989	0.011
PARK2	985	0.069
FGFR3	983	0.099
WT1	983	0.044
TLR4	983	0.147
TLR2	982	0.119
MET	981	0.098
APC	976	0.131
PDGFRB	976	0.23
KIT	971	0.123
KDR	966	0.225
ERBB2	966	0.283
BAX	962	0.208
IGF2R	962	0.06
KRAS	960	0.179
RUNX1	958	0.194
PDGFB	958	0.07
NTRK1	955	0.212
IL1RN	954	0.118
BACH1	954	0.02

NME1	953	0.052
PTEN	953	0.26
TGFBR2	948	0.189
HNF1B	947	0.044
NF1	947	0.085
RET	932	0.284
BRCA1	932	0.518
PRKAR1A	925	0.055
AXIN2	920	0.096
BRCA2	920	0.255
CYP2A6	917	0.209
TERT	916	0.369
MAX	913	0.249
PAX7	903	0.077
MSH2	899	0.191
PIK3CA	899	0.859
ZFHX3	899	0.285
ARHGAP26	899	0.12
BCR	899	0.422
GMPS	899	0.396

MAP3K8	896	0.095
MCC	895	0.016
NRAS	891	0.486
CASP8	889	0.389
AURKA	883	0.634
TSG101	878	0.176
PTPN12	877	0.116
GDNF	863	0.383
ETV6	857	0.157
STK11	856	0.348
CDKN2A	855	0.48
MLH1	852	0.267
GATA1	846	0.426
BRAF	845	0.381
HMMR	838	0.341
IRF4	837	0.243
HNF1A	836	0.349
NPM1	827	0.508
GATA2	826	0.222
RASSF1	826	0.224

ARHGEF12	822	0.184
GPC3	816	0.101
NUP214	812	0.526
PAX3	800	0.384
CD82	791	0.232
ODC1	786	0.268
MUTYH	784	0.084
NCOA4	776	0.131
MSR1	767	0.16
FLT3	764	0.412
NQO2	744	0.035
OGG1	736	0.304
XRCC3	718	0.262
ATM	714	0.492
FLCN	714	0.106
KLF6	714	0.119
LIG4	706	0.31
WWOX	706	0.156
RNASEL	698	0.097
PTPRJ	682	0.144

PLAG1	677	0.167
RASA1	645	0.611
PDGFRL	625	0.139
PDGFRA	612	0.746
DLC1	605	0.257
NF2	603	0.481
SDHC	589	0.313
DLEC1	571	0.351
SMARCE1	563	0.408
CHIC2	556	0.136
MLF1	550	0.238
SDHB	548	0.631
RAD54L	546	0.156
PCM1	538	0.284
CBFB	511	0.416
PTCH2	507	0.389
PTCH1	497	0.638
BUB1B	489	0.914
CASP10	481	0.592
FLT4	473	0.562

ANTXR1	470	0.218
MXI1	460	0.492
SUFU	457	0.636
LZTS1	424	0.545
TRIM24	419	0.513
MAD1L1	419	0.787
SH3GL1	417	0.498
PPP2R1B	413	0.64
BARD1	411	0.767
IDH1	409	0.698
EXT1	404	0.458
MSH6	403	0.834
RB1CC1	401	0.652
ERCC6	391	0.477
PRCC	385	0.385
HIP1	384	0.675
PALB2	383	0.45
RNF6	374	0.306
DIRC2	364	0.405
BRIP1	363	0.527

PPM1D	350	0.77
CHEK2	342	0.97
CCDC6	333	0.55
SDHD	333	0.87
KIF1B	329	0.709
FH	328	0.868
MLH3	312	0.67
ASPSCR1	309	0.73
SH2D1A	294	0.791
DCC	291	0.712
TACC3	286	0.826
BLM	286	0.857
RNF139	272	0.75
WHSC1L1	257	0.438
MN1	247	0.623
PHB	246	0.965
PMS2	218	0.804
CDS1	212	0.409
LPP	188	0.776
ATR	159	0.988

GOLGA5	0	1
TMEM127	0	1
FEZ1	0	1
RAD54B	0	1
TRIM33	0	1
MINPP1	0	1
COL4A6	0	1

3. Genes related to cardiovascular

Gene	Maximum interaction score	Permutation FDR
BMPR2	999	0.039
DSP	999	0.009
IL6	999	0.042
PPARG	999	0.017
ESR1	998	0.138
F2	998	0.076
F5	998	0.036
TCF7L2	997	0.029
EPHX1	995	0.012
TCF4	994	0.028
ELN	993	0.033

PKP2	984	0.024
NOTCH3	983	0.065
NKX2-5	978	0.052
GATA4	977	0.051
PTGIS	977	0.011
F13A1	970	0.037
HNF4A	963	0.125
NOS3	963	0.16
HGF	961	0.106
GHR	959	0.118
LDLR	958	0.09
GJA1	953	0.097
NR3C2	953	0.029
PNMT	952	0.04
KCNQ1	951	0.138
MYH7	948	0.076
HNF1B	947	0.044
NOS2	947	0.096
OLR1	944	0.018
NEUROD1	939	0.035

CYP3A5	934	0.103
AGTR1	928	0.094
PRKAR1A	925	0.055
MEF2A	923	0.126
GNAI2	918	0.399
TPM1	918	0.292
APOA2	910	0.168
GCGR	907	0.157
SELE	907	0.089
ADRB1	901	0.191
DES	901	0.338
HMGA1	899	0.31
LMNA	899	0.501
MTNR1B	899	0.666
TNNI3	880	0.262
GCLC	872	0.146
IRS1	866	0.565
CAV3	863	0.188
TNFSF4	859	0.042
GCLM	855	0.135

KRIT1	854	0.412
PTPN1	852	0.386
PRKAG2	837	0.18
HNF1A	836	0.349
SCN5A	836	0.121
SLC6A2	834	0.05
NAT1	832	0.081
GCK	829	0.272
MAPK8IP1	826	0.188
GATA6	823	0.181
SLC2A4	810	0.527
IRS2	802	0.497
AKT2	800	0.543
ENG	800	0.267
RETN	800	0.193
TCAP	800	0.301
WFS1	787	0.333
LIPH	786	0.089
MYBPC3	786	0.279
EPHX2	784	0.071

GPD2	783	0.189
LIPC	778	0.38
GIP	776	0.14
ALOX5AP	770	0.095
JAG1	754	0.292
ANK2	750	0.211
ZFPM2	750	0.126
CDKAL1	748	0.138
DMD	747	0.575
ABCC8	708	0.29
ITIH4	694	0.164
TEK	681	0.35
AGT	664	0.317
ATP1B1	661	0.098
KCNJ11	655	0.398
NET1	647	0.107
RASA1	645	0.611
PDE4D	624	0.462
ADD1	619	0.475
KCNJ2	612	0.34

PAX4	609	0.227
LRP8	599	0.468
RYR2	593	0.394
GDF1	582	0.173
RGS5	577	0.243
LTA	561	0.646
ABCA1	556	0.671
PSMA6	530	0.695
PRKCH	525	0.354
MYL2	447	0.606
GNB3	411	0.607
IGF2BP2	407	0.431
ENPP1	386	0.671
CSRP3	380	0.606
TBX1	364	0.474
FH	328	0.868
EYA4	323	0.385
ECE1	319	0.506
KCNMB1	302	0.608
ABCC9	294	0.504

F7	294	0.52
SGCD	291	0.701
SCO2	262	0.704
MYL3	246	0.79
DTNA	234	0.704
LGALS2	199	0.47
MYLK2	183	0.666
CFC1	0	1
NKX2-6	0	1

4. Genes related to connective tissue disorder

Gene	Maximum interaction score	Permutation FDR
IL6	999	0.042
ELN	993	0.033
IL10	982	0.119
SCT	979	0.07
HGF	961	0.106
COL1A2	930	0.316
COL1A1	927	0.322
TGFBR1	907	0.334
ANTXR2	905	0.007

HLA-DRB1	899	0.283
SOS1	899	0.717
FGFR2	878	0.33
PTPN22	868	0.063
FRZB	825	0.077
CD244	786	0.141
MIF	724	0.192
PADI4	708	0.194
ANKH	690	0.129
B4GALT7	651	0.066
FBN1	633	0.319
ADAMTS2	567	0.144
COL3A1	565	0.629
PLOD1	497	0.425
KRT13	491	0.194
KRT4	491	0.402
FBLN5	486	0.183
COL5A1	462	0.616
GNPAT	460	0.342
SLC22A4	436	0.504

COL5A2	435	0.613
NFKBIL1	407	0.453
ENPP1	386	0.671
ASPN	374	0.473
XYLT2	368	0.252
SLC26A2	353	0.368
HLA-B	334	0.617
ARSE	321	0.349
XYLT1	310	0.297
ADAMTS10	308	0.079
COL6A1	304	0.661
FLNB	296	0.634
COL11A2	294	0.063
ABCC6	241	0.703
EBP	168	0.821
SMARCAL1	0	1
TNXB	0	1

5. Genes related to dermatological

Gene	Maximum interaction score	Permutation FDR
DSP	999	0.009

DSG1	996	0.009
MMP1	995	0.032
EDAR	993	0.023
EDA	965	0.034
ALOX12B	964	0.009
SLURP1	945	0.009
IKBKG	940	0.373
KRT5	918	0.079
CYLD	916	0.08
KRT16	916	0.078
KRT10	914	0.048
KRT14	914	0.108
KRT9	913	0.014
ERCC3	909	0.339
KRT2	905	0.095
KRT1	904	0.115
KRT6A	901	0.141
KRT86	900	0.13
ERCC2	899	0.647
ERCC5	899	0.528

HLA-C	899	0.389
LMNA	899	0.501
PKP1	899	0.226
PLEC	886	0.099
SAT1	854	0.094
PVRL1	819	0.234
TGM1	817	0.095
RAB27A	808	0.216
TYR	803	0.228
GJB2	766	0.124
NOD2	747	0.378
CDSN	734	0.083
FLCN	714	0.106
ERCC4	643	0.44
GJB6	632	0.163
SOX18	616	0.175
ITGA6	611	0.736
PSTPIP1	605	0.223
MLPH	587	0.232
CDH3	578	0.141

DSG4	569	0.123
LTA	561	0.646
DDB2	536	0.424
ITGB4	522	0.784
KRT17	480	0.249
SPINK5	480	0.315
GJB3	473	0.247
LAMB3	452	0.534
MYO5A	443	0.406
HLA-A	424	0.294
KRT81	390	0.25
ADAR	382	0.437
ABCA12	379	0.276
LAMA3	365	0.58
LAMC2	361	0.512
ZMPSTE24	348	0.718
ATP2C1	335	0.415
COL17A1	334	0.542
HLA-B	334	0.617
MBTPS2	313	0.436

MPLKIP	301	0.254
KRT6B	293	0.448
COL7A1	281	0.61
TMC6	247	0.382
DKC1	234	0.998
SLC39A4	159	0.181
TMC8	157	0.437
GJB4	154	0.222
HR	0	1
KRT83	0	1
POLH	0	1

6. Genes related to developmental

Gene	Maximum interaction score	Permutation FDR
IGF1	999	0.069
PTPN11	999	0.098
ZEB2	993	0.007
AMH	985	0.039
NKX2-5	978	0.052
CYP1B1	969	0.119
TWIST1	962	0.056

CCL2	961	0.27
SHH	956	0.362
VANGL1	953	0.056
LHCGR	927	0.188
INSR	903	0.438
PITX2	899	0.394
FGFR2	878	0.33
MTHFR	854	0.156
GATA6	823	0.181
MECP2	816	0.315
AMHR2	810	0.069
TBX5	804	0.177
MSX1	804	0.264
MKKS	801	0.13
IRF6	800	0.044
TNNI2	778	0.252
PAX6	760	0.48
EZH2	746	0.362
MIF	724	0.192
MTRR	694	0.19

NODAL	591	0.168
HSD17B3	591	0.279
TPM2	585	0.401
GDF1	582	0.173
TSC1	575	0.743
CDKL5	524	0.261
SIX3	514	0.492
MTR	504	0.419
TBX22	491	0.144
TNNT3	443	0.453
T	400	0.299
ZIC2	377	0.519
TBX1	364	0.474
UBE3A	353	0.837
LEMD3	330	0.528
MYH3	308	0.465
NIPBL	272	0.588
ESCO2	255	0.643
NSDHL	229	0.519
TRPS1	221	0.562

TCOF1	218	0.697
ATR	159	0.988
CFC1	0	1
MED13L	0	1
FUZ	0	1
NKX2-6	0	1
EZH1	0	1

7. Genes related to ear nose throat

Gene	Maximum interaction score	Permutation FDR
MYO7A	951	0.069
MYH14	950	0.224
MYO6	899	0.61
SLC26A4	881	0.042
OTOF	873	0.033
MYO15A	852	0.017
CDH23	799	0.087
WFS1	787	0.333
TECTA	773	0.027
TMC1	770	0.025
TMIE	766	0.014

GJB2	766	0.124
KCNQ4	752	0.046
TMPRSS3	728	0.035
GRHL2	718	0.026
ACTG1	686	0.601
DSPP	673	0.275
PCDH15	657	0.244
DIAPH1	641	0.303
GJB6	632	0.163
COCH	627	0.126
POU3F4	625	0.132
MYH9	538	0.462
KCNJ10	536	0.177
POU4F3	491	0.264
FOXI1	490	0.258
USH1C	473	0.274
GJB3	473	0.247
OTOA	452	0.134
STRC	427	0.167
MYO3A	360	0.329

ESPN	338	0.338
CTSC	335	0.673
EYA4	323	0.385
COL11A2	294	0.063
ATP2B2	257	0.586
DFNB31	251	0.526
MYO1A	0	1

8. Genes related to endocrine

Gene	Maximum interaction score	Permutation FDR
IL6	999	0.042
AIP	999	0.01
PPARG	999	0.017
AR	999	0.073
TCF7L2	997	0.029
TCF4	994	0.028
GNAS	966	0.054
GATA3	964	0.068
HNF4A	963	0.125
HGF	961	0.106
NR3C2	953	0.029

FOXP3	951	0.215
STAT5B	950	0.149
HNF1B	947	0.044
NEUROD1	939	0.035
OAS1	931	0.056
HESX1	923	0.027
BMP15	922	0.006
GCGR	907	0.157
THRB	906	0.251
PTH	902	0.425
GNRHR	899	0.615
MTNR1B	899	0.666
SSTR5	899	0.668
CASR	899	0.857
TSHR	899	0.484
HMGA1	899	0.31
MC2R	899	0.408
AVPR2	899	0.416
FSHR	899	0.456
SCNN1A	898	0.043

SCNN1B	887	0.077
SCNN1G	878	0.077
PTPN22	868	0.063
IRS1	866	0.565
PTPN1	852	0.386
TG	848	0.146
FSHB	845	0.233
PAX8	845	0.127
HNF1A	836	0.349
GCK	829	0.272
TPO	829	0.144
MAPK8IP1	826	0.188
DHH	826	0.084
SLC5A5	823	0.129
AVP	822	0.197
SLC2A4	810	0.527
IRS2	802	0.497
RETN	800	0.193
AKT2	800	0.543
CTLA4	793	0.455

WFS1	787	0.333
STAR	786	0.286
LIPH	786	0.089
GPD2	783	0.189
LIPC	778	0.38
CAPN10	772	0.046
CYP11B1	764	0.209
CDKAL1	748	0.138
CYP17A1	719	0.395
STX16	709	0.234
ABCC8	708	0.29
PPP1R3A	669	0.308
SUMO4	657	0.165
KCNJ11	655	0.398
PAX4	609	0.227
TBX19	585	0.14
CACNA1S	535	0.395
FOXE1	518	0.404
ITPR3	504	0.516
PROP1	480	0.315

TTF2	472	0.287
TBCE	424	0.446
IGF2BP2	407	0.431
AQP2	405	0.55
ENPP1	386	0.671
SYCP3	276	0.596
MRAP	238	0.803
PTF1A	159	0.693
FGD2	0	1

9. Genes related to gastrointestinal

Gene	Maximum interaction score	Permutation FDR
IL6	999	0.042
EPHX1	995	0.012
UGT1A1	977	0.026
CFTR	966	0.108
HGF	961	0.106
ABCB4	936	0.024
RET	932	0.284
KRT18	912	0.084
KRT8	904	0.16

BAAT	899	0.088
EDNRB	899	0.668
FGF10	884	0.168
TJP2	778	0.312
NOD2	747	0.378
ATP8B1	694	0.149
CIRH1A	692	0.017
SLC26A3	641	0.259
VPS33B	518	0.2
SPINK1	382	0.455
ABCB11	365	0.506
PRKCSH	358	0.379
PRSS1	294	0.686
SEC63	259	0.583
CTRC	190	0.792
HSD3B7	0	1

10. Genes related to hematological

Gene	Maximum interaction score	Permutation FDR
FGA	999	0.02
JAK2	999	0.116

VHL	998	0.052
IFNG	997	0.076
FGB	997	0.018
GGCX	990	0.031
APOE	990	0.114
TF	978	0.039
ITGA2B	976	0.066
EPOR	967	0.15
RUNX1	958	0.194
CD36	945	0.11
ELANE	943	0.169
CYP2C9	942	0.179
CYP2A6	917	0.209
NT5C3A	899	0.505
ACHE	899	0.079
GCLC	872	0.146
GATA1	846	0.426
LMAN1	844	0.069
TPO	829	0.144
THPO	825	0.211

CR1	801	0.201
SH2B3	800	0.171
VKORC1	786	0.05
SLC4A1	778	0.293
RPS14	730	0.713
A4GALT	726	0.06
GP1BB	694	0.109
HBB	681	0.246
CP	651	0.516
CPN1	619	0.125
CFH	615	0.291
PDGFRA	612	0.746
BSG	604	0.387
FOXC2	595	0.41
GP1BA	589	0.317
AQP1	569	0.137
GCNT2	565	0.397
MPL	544	0.523
MYH9	538	0.462
CFHR1	526	0.194

CFHR3	510	0.167
ADAMTS13	491	0.143
ATRX	484	0.589
ANKRD26	480	0.218
FLT4	473	0.562
GSS	470	0.425
EPX	465	0.256
CDAN1	436	0.169
AQP3	334	0.541
RPS19	323	0.918
MASTL	319	0.172
CUBN	274	0.591
SLC19A2	273	0.289
F9	266	0.656
WAS	213	0.971
GIF	202	0.57
RHAG	202	0.578
AMN	180	0.471
GP9	151	0.793
UNC13D	0	1

ABCB7	0	1
LBR	0	1
FHL3	0	1

11. Genes related to immunological

Gene	Maximum interaction score	Permutation FDR
CXCR1	999	0.022
IFNGR1	999	0.017
IL6	999	0.042
STAT1	999	0.104
IFNG	997	0.076
IL12RB1	997	0.053
JAK3	996	0.084
IL12B	993	0.093
TNFRSF1A	993	0.147
CXCR4	991	0.064
TAP1	988	0.039
PARK2	985	0.069
CX3CR1	984	0.028
IL10	982	0.119
TLR2	982	0.119

CISH	968	0.04
CXCL12	966	0.151
MPO	966	0.027
IL13	964	0.145
CD40	963	0.289
CCL2	961	0.27
CD40LG	961	0.162
HGF	961	0.106
TLR5	950	0.045
CD8A	939	0.461
TIRAP	939	0.102
BTK	922	0.291
IFNGR2	922	0.12
TLR3	913	0.332
PLA2G7	910	0.137
CCL11	909	0.219
ITGB2	909	0.346
RAC2	907	0.385
HLA-C	899	0.389
HLA-DRB1	899	0.283

IL21R	899	0.05
MC3R	899	0.395
MEFV	899	0.234
RFXANK	899	0.285
CASP8	889	0.389
IL4R	882	0.313
AICDA	880	0.201
PTPN22	868	0.063
DNASE1	858	0.01
FCGR2B	837	0.098
CEBPE	834	0.112
FAS	824	0.331
PTPRZ1	819	0.111
SELP	814	0.326
AIRE	800	0.128
CCL3	800	0.216
BANK1	780	0.042
RAG2	771	0.262
C4A	730	0.101
SP110	716	0.05

ATM	714	0.492
NLRP3	712	0.319
LIG4	706	0.31
CD209	670	0.346
KIR3DL1	578	0.283
NCF1	577	0.446
RAG1	561	0.502
CYBA	509	0.382
CASP10	481	0.592
SPINK5	480	0.315
MRE11A	414	0.772
CYBB	405	0.516
SLC11A1	377	0.546
NCF2	367	0.685
PHF11	360	0.492
TNFRSF13B	360	0.796
RFX5	353	0.408
KIR3DS1	352	0.592
ICOS	346	0.768
HAVCR1	324	0.702

SERPING1	310	0.57
ADA	307	0.821
UNG	265	0.838
MS4A2	234	0.621
WAS	213	0.971
DCLRE1C	183	0.807
MVK	160	0.823
RFXAP	0	1
TAP2	0	1
TAPBP	0	1

12. Genes related to metabolic

Gene	Maximum interaction score	Permutation FDR
APOA1	999	0.038
MCCC2	999	0.007
PPARG	999	0.017
OTC	995	0.035
SLC40A1	993	0.009
GBA	978	0.021
UGT1A1	977	0.026
PCCB	971	0.027

ATP7B	963	0.02
SMPD1	962	0.043
GHR	959	0.118
LDLR	958	0.09
SLC2A1	954	0.066
ASS1	952	0.064
GLUD1	952	0.041
CTH	947	0.033
ALG2	945	0.02
ALG3	937	0.023
DBH	935	0.071
PDX1	932	0.205
ASL	930	0.055
IVD	930	0.027
LCAT	929	0.022
ECM1	922	0.014
GAA	917	0.051
ACADM	910	0.15
APOA2	910	0.168
AUH	910	0.051

SLC2A2	908	0.048
USF1	900	0.228
ADSL	899	0.318
ALDH3A2	899	0.317
ALDH4A1	899	0.132
AMT	899	0.199
HAL	899	0.146
HPRT1	899	0.4
LMNA	899	0.501
HFE	884	0.112
CAV3	863	0.188
GK	859	0.062
HLCS	854	0.042
MTHFR	854	0.156
NPC1	854	0.027
PMM2	849	0.095
SCD	842	0.08
ABCG8	839	0.066
ABCG5	836	0.065
APOA5	836	0.134

APOB	836	0.337
CYP27A1	833	0.132
GCK	829	0.272
CETP	825	0.11
ATIC	821	0.34
CYP11B2	820	0.133
TFR2	805	0.101
SLC5A1	801	0.06
ASPA	800	0.202
L2HGDH	800	0.073
PRODH	800	0.132
WFS1	787	0.333
EPHX2	784	0.071
ETFA	784	0.326
FMO3	782	0.056
HYAL1	782	0.03
ALPL	779	0.182
BTD	766	0.053
DDC	762	0.338
CPT1A	750	0.212

HEXA	746	0.195
NAGS	736	0.132
PHGDH	734	0.136
NPC2	724	0.062
ABCC8	708	0.29
GYS2	702	0.302
ACAT1	698	0.356
ITIH4	694	0.164
MTRR	694	0.19
PPP1R3A	669	0.308
H6PD	663	0.256
HMGCS2	657	0.204
CP	651	0.516
GNE	649	0.12
ETFB	641	0.2
ALG1	629	0.19
DBT	622	0.332
ACADVL	591	0.414
GBE1	587	0.197
HSD17B4	567	0.167

ALG6	563	0.199
B4GALT1	563	0.322
PCSK9	563	0.35
MUT	562	0.433
GNPTAB	561	0.037
ABCA1	556	0.671
ARG1	553	0.373
ABCC2	542	0.295
GNMT	523	0.283
MPDU1	523	0.185
PANK2	520	0.128
SLC25A13	506	0.088
MTR	504	0.419
PCCA	488	0.332
GNS	486	0.174
SLC6A19	484	0.211
MPI	483	0.471
GSS	470	0.425
OPA3	469	0.092
DHCR24	462	0.374

SUMF1	455	0.219
MADD	452	0.565
SLC25A15	445	0.168
MGAT2	443	0.284
AGXT	434	0.505
GCSH	433	0.414
NAGA	405	0.364
LAMP2	389	0.645
MAN2B1	389	0.306
NEU1	386	0.541
BCKDHB	384	0.644
SLC7A7	380	0.271
DPAGT1	369	0.453
PC	367	0.753
ALG12	366	0.249
GALT	350	0.566
RP1	348	0.673
LDLRAP1	341	0.59
IDUA	340	0.579
MCOLN1	332	0.376

ETFDH	328	0.515
FH	328	0.868
HGD	327	0.283
FTCD	324	0.366
GALK1	324	0.579
TALDO1	324	0.575
ETHE1	323	0.441
SLC22A5	319	0.606
ALG8	307	0.26
LIPI	307	0.266
MMAA	302	0.377
ABCD1	301	0.597
BCKDHA	291	0.694
MMAB	291	0.471
GCDH	288	0.456
HSD17B10	285	0.871
MCM6	279	0.835
AGPAT2	278	0.696
PHEX	269	0.805
BSCL2	267	0.719

BCS1L	258	0.813
PYGM	256	0.859
GLDC	251	0.563
ACADS	238	0.658
CPT2	213	0.709
SUOX	201	0.404
CPS1	190	0.746
MLYCD	182	0.537
AMN	180	0.471
XDH	172	0.863
DPM1	168	1
SLC17A5	168	0.762
PPOX	160	0.776
UROS	157	0.474
HEXB	154	0.892
AASS	0	1
ABHD5	0	1
COG7	0	1
D2HGDH	0	1
DMGDH	0	1

GRHPR	0	1
MANBA	0	1
MCCC1	0	1
MOCS1	0	1
OCRL	0	1
PHKB	0	1
RPIA	0	1
SGSH	0	1
SLC35C1	0	1

13. Genes related to multiple

Gene	Maximum interaction score	Permutation FDR
LIFR	999	0.012
JUP	999	0.068
PTPN11	999	0.098
DSP	999	0.009
NOG	998	0.055
KCNJ1	997	0.01
CREBBP	995	0.124
HRAS	992	0.125
COL2A1	990	0.028

EGR2	985	0.036
WT1	983	0.044
ATP7A	983	0.014
SNAI2	978	0.007
UGT1A1	977	0.026
GNAS	966	0.054
BACH1	954	0.02
GFAP	953	0.059
PTEN	953	0.26
KCNQ1	951	0.138
FOXP3	951	0.215
MITF	948	0.188
MYOC	947	0.015
DNMT3B	945	0.068
PAX2	939	0.142
LOR	936	0.082
MGP	934	0.036
FOXL2	929	0.019
WRN	927	0.155
PRKAR1A	925	0.055

HESX1	923	0.027
BRCA2	920	0.255
INSR	903	0.438
PITX2	899	0.394
MID1	899	0.243
EDNRB	899	0.668
FGFR1	899	0.393
FGFR2	878	0.33
GATA1	846	0.426
TBX3	822	0.069
BBS2	816	0.158
GPC3	816	0.101
MYCN	815	0.352
ZIC3	815	0.059
WNT3	807	0.2
TYR	803	0.228
MKKS	801	0.13
IRF6	800	0.044
PAX3	800	0.384
GRIP1	800	0.29

CDH23	799	0.087
BBS4	793	0.164
LMX1B	784	0.198
GFM1	780	0.221
SLC12A1	768	0.167
GJB2	766	0.124
SMS	756	0.143
JAG1	754	0.292
NOD2	747	0.378
RPS6KA3	742	0.359
SKI	729	0.186
ROR2	724	0.105
NLRP3	712	0.319
LIG4	706	0.31
CLDN1	704	0.275
CDKN1C	699	0.438
CACNA1C	670	0.419
FLNA	663	0.592
BSND	663	0.12
PCDH15	657	0.244

RASA1	645	0.611
L1CAM	629	0.454
NDUFB3	615	0.424
FANCA	609	0.549
BBS12	595	0.16
AHI1	591	0.205
TFAP2B	585	0.219
BBS7	581	0.215
PEX7	577	0.192
TSC1	575	0.743
KIAA1279	563	0.144
AGPS	552	0.132
NDUFV2	550	0.51
MYH9	538	0.462
SALL4	529	0.32
TRIM32	510	0.402
PTCH1	497	0.638
RAB3GAP1	488	0.16
ATRX	484	0.589
NDUFS1	480	0.545

NDUFV1	480	0.622
MKS1	477	0.434
COL11A1	475	0.579
CHD7	474	0.322
USH1C	473	0.274
FREM2	470	0.124
HSPG2	452	0.803
CEP290	445	0.676
NDUFS3	435	0.792
TP63	433	0.744
NDN	430	0.622
TBCE	424	0.446
TAZ	417	0.579
USH2A	412	0.43
TYMP	401	0.587
ERCC6	391	0.477
SDS	389	0.317
HOXA13	386	0.385
SALL1	385	0.404
ATPAF2	368	0.158

LAMA3	365	0.58
TBX1	364	0.474
BRIP1	363	0.527
PRX	357	0.549
BCOR	351	0.424
ZMPSTE24	348	0.718
MPZ	344	0.448
CLCNKB	340	0.293
ARX	339	0.582
PMP22	337	0.63
CTSC	335	0.673
LEMD3	330	0.528
SDHA	312	0.848
POMGNT1	312	0.441
HOXA11	307	0.604
SEMA3E	302	0.21
CRLF1	296	0.427
COL11A2	294	0.063
TRIM37	293	0.428
NDUFS2	283	0.876

BBS5	277	0.43
VPS13B	274	0.426
FRAS1	272	0.306
LYST	262	0.737
COL18A1	260	0.818
BCS1L	258	0.813
USH1G	246	0.405
HPS1	246	0.759
DHCR7	243	0.848
CHM	234	0.792
NDUFB9	217	0.818
NDUFA1	215	0.911
CUL7	213	0.737
AAAS	208	0.781
POMT1	208	0.669
DNAI1	190	0.796
DGUOK	185	0.796
NDUFS4	172	0.851
FANCB	151	0.927
BBS9	0	1

SBDS	0	1
PEX1	0	1
AP3B1	0	1
CFC1	0	1
NDUFAF1	0	1
FOXRED1	0	1
ERCC8	0	1
DYM	0	1
NDUFS6	0	1
NUBPL	0	1
NDUFAF2	0	1
NDUFA11	0	1
CLRN1	0	1
RAI1	0	1
NDUFAF3	0	1
PHF6	0	1
ARL6	0	1
NDUFAF4	0	1
PDZD7	0	1
INPP5E	0	1

CCDC28B	0	1
PUS1	0	1
NDUFAF5	0	1
SDHAF1	0	1
TTC8	0	1
BBS10	0	1
KIF7	0	1
GPR98	0	1
LCA10	0	1
TMEM67	0	1

14. Genes related to muscular

Gene	Maximum interaction score	Permutation FDR
AR	999	0.073
SMAD1	966	0.163
MYH7	948	0.076
MUSK	944	0.105
DES	901	0.338
CHRNE	899	0.028
LMNA	899	0.501
CAPN3	893	0.123

PLEC	886	0.099
CAV3	863	0.188
SGCA	826	0.17
CRYAB	814	0.104
TCAP	800	0.301
EMD	800	0.196
CHAT	778	0.395
SGCB	760	0.25
DMD	747	0.575
ACTA1	726	0.494
DNM2	714	0.653
SCN4A	706	0.087
SGCG	681	0.204
GNE	649	0.12
NEB	621	0.424
VCP	590	0.67
DMPK	532	0.425
RYR1	515	0.563
TRIM32	510	0.402
SCG3	491	0.3

ATP2A1	462	0.331
LARGE	413	0.289
PABPN1	390	0.8
SMN2	385	0.634
VAPB	381	0.506
CLCN1	374	0.414
SEPN1	374	0.472
CHRNB1	362	0.29
COL6A3	354	0.622
COL6A2	339	0.575
SMN1	334	0.698
LAMA2	331	0.755
IGHMBP2	311	0.424
COL6A1	304	0.661
SGCD	291	0.701
MYOT	270	0.628
BSCL2	267	0.719
RAPSN	249	0.642
DYSF	249	0.752
FKTN	229	0.681

MTM1	223	0.902
CPT2	213	0.709
POMT1	208	0.669
DGUOK	185	0.796
TNNT1	162	0.81
MYH2	0	1
CHRND	0	1
CHRNA1	0	1
MTMR14	0	1
FKRP	0	1
GARS	0	1

15. Genes related to neurological

Gene	Maximum interaction score	Permutation FDR
APOA1	999	0.038
FGA	999	0.02
TBP	999	0.191
ESR1	998	0.138
PSEN1	997	0.073
PLAU	996	0.032
PRKCG	995	0.01

TNF	993	0.301
APOE	990	0.114
EGR2	985	0.036
PARK2	985	0.069
ATP7A	983	0.014
GBA	978	0.021
APTX	975	0.025
MPO	966	0.027
SMAD1	966	0.163
NOS3	963	0.16
GJB1	961	0.026
SNCA	960	0.199
DRD2	956	0.082
SHH	956	0.362
NTRK1	955	0.212
SPTLC1	946	0.05
HSPB1	944	0.105
NDRG1	943	0.031
RET	932	0.284
FXN	931	0.046

NKX2-1	925	0.165
APP	918	0.579
LYZ	911	0.057
ARHGEF6	899	0.176
DCX	899	0.198
EDNRA	899	0.701
GALC	899	0.236
HLA-DRB1	899	0.283
IKBKAP	899	0.481
LRPPRC	899	0.054
MAPT	899	0.351
OPHN1	899	0.134
PAFAH1B1	899	0.813
SYN1	898	0.126
SPG7	894	0.056
PER2	892	0.151
HFE	884	0.112
HLA-DQB1	864	0.092
KRIT1	854	0.412
HCFC1	843	0.449

ACE	834	0.176
FTL	834	0.137
CLN3	833	0.092
SGCE	827	0.144
ATL1	826	0.062
RELN	825	0.359
PSEN2	820	0.503
WNK1	817	0.086
HOXA1	816	0.029
MECP2	816	0.315
PLP1	815	0.067
SOD1	815	0.345
ATN1	800	0.172
CSTB	800	0.099
SLC6A8	767	0.058
PHYH	762	0.118
SMS	756	0.143
ARFGEF2	755	0.104
PARK7	753	0.25
FOXP2	750	0.195

CACNA1A	724	0.317
PDC	715	0.649
DNM2	714	0.653
PDCD1	712	0.264
SCN4A	706	0.087
COL9A2	696	0.084
KIF5A	696	0.217
LITAF	690	0.061
PAK3	683	0.334
FLNA	663	0.592
SLC16A2	659	0.105
IGBP1	655	0.518
KCNMA1	655	0.385
CP	651	0.516
KCNQ2	651	0.3
PPT1	643	0.212
NDUFA9	635	0.463
L1CAM	629	0.454
LRRK2	605	0.251
KCNQ3	599	0.316

CST3	595	0.331
HSPD1	591	0.746
SOX10	591	0.491
NEFH	589	0.168
SLC19A3	579	0.065
GPR56	553	0.125
PPP2R2B	543	0.427
COL9A3	541	0.143
PINK1	536	0.325
CACNA1S	535	0.395
GLRA1	520	0.148
PANK2	520	0.128
SORL1	519	0.358
CLN8	518	0.158
HSPB8	518	0.408
RYR1	515	0.563
SIX3	514	0.492
APBB2	508	0.255
COX15	488	0.362
GABRG2	488	0.262

FGF14	484	0.312
GCH1	478	0.71
COL11A1	475	0.579
SCN1A	466	0.331
CHRNB2	462	0.361
HSPG2	452	0.803
ATP1A3	446	0.293
CLN6	445	0.192
ROBO3	441	0.151
NDUFS3	435	0.792
NDUFS8	432	0.708
CLN5	429	0.188
THBS2	420	0.625
DCTN1	416	0.88
PRPH	401	0.487
CHRNA4	396	0.521
SACS	396	0.313
BLMH	393	0.474
TOR1A	393	0.646
COL4A1	383	0.861

VAPB	381	0.506
ASPN	374	0.473
MFN2	367	0.564
EYA1	366	0.621
PNKD	360	0.248
SURF1	358	0.409
VLDLR	358	0.717
SCN1B	355	0.41
EIF2B2	353	0.365
ITM2B	353	0.49
MPZ	344	0.448
MR1	340	0.321
ARX	339	0.582
PMP22	337	0.63
TTF1	337	0.527
TPP1	330	0.471
KIF1B	329	0.709
MCPH1	328	0.421
SLC12A6	327	0.524
COLQ	326	0.384

PQBP1	323	0.505
NLGN3	321	0.452
SPG21	318	0.462
JPH3	312	0.529
SDHA	312	0.848
GAN	308	0.509
CCM2	305	0.446
PEX5	303	0.459
ABCD1	301	0.597
SCN9A	299	0.39
TIMM8A	299	0.484
SCN2A	297	0.719
SLC25A22	296	0.133
NIPA1	294	0.415
SH3TC2	294	0.376
SPG20	294	0.612
SNCB	290	0.53
ATXN1	286	0.779
SOX3	285	0.674
ATXN2	281	0.829

GSN	280	0.97
EIF2B1	277	0.727
EIF2B3	277	0.383
EIF2B4	277	0.268
EIF2B5	277	0.458
SNAP29	271	0.566
BSCL2	267	0.719
BCS1L	258	0.813
ATXN7	255	0.817
MLC1	255	0.766
ATXN3	254	0.884
SPAST	254	0.653
ARHGEF10	252	0.444
SETX	243	0.764
PDHA1	238	0.778
CHM	234	0.792
ALS2	232	0.706
SLC25A19	230	0.513
NHLRC1	227	0.569
EPM2A	221	0.703

POLG	217	0.81
TTPA	210	0.599
ATP1A2	196	0.785
CILP	196	0.382
VPS13A	193	0.567
AMN	180	0.471
SERPINI1	180	0.66
ASPM	173	0.962
NDUFS4	172	0.851
PRSS12	172	0.333
GJC2	171	0.409
KDM5C	167	0.882
CRBN	160	0.632
ACSL4	0	1
ALMS1	0	1
ARSA	0	1
ATCAY	0	1
CDK5RAP2	0	1
CENPJ	0	1
CLCN2	0	1

EFHC1	0	1
FOXRED1	0	1
FTSJ1	0	1
GARS	0	1
GDAP1	0	1
GPR98	0	1
LGI1	0	1
ME2	0	1
MTMR2	0	1
NDUFA10	0	1
NDUFA12	0	1
NDUFA2	0	1
NDUFAF2	0	1
NDUFAF6	0	1
NDUFS7	0	1
PEX12	0	1
SBF2	0	1
TDP1	0	1
ZNF81	0	1

16. Genes related to nutritional

Gene	Maximum interaction score	Permutation FDR
PPARG	999	0.017
POMC	996	0.093
PYY	995	0.02
UCP1	991	0.011
GHRL	960	0.085
AGRP	958	0.096
NR0B2	941	0.174
ADRB2	918	0.194
MC4R	913	0.141
HTR2A	907	0.13
ADRB3	899	0.427
UCP3	863	0.094
SLC6A14	530	0.131
SIM1	472	0.421
PPARGC1B	464	0.555
PCSK1	436	0.455
ENPP1	386	0.671
SDC3	367	0.699

17. Genes related to ophthamological

Gene	Maximum interaction score	Permutation FDR
LRP5	998	0.024
APOE	990	0.114
PLG	984	0.09
NDP	976	0.022
CYP1B1	969	0.119
DRD5	961	0.014
TGFBI	953	0.087
MYOC	947	0.015
SOX2	939	0.154
PITX3	909	0.032
KRT3	904	0.08
GUCY2D	899	0.512
PITX2	899	0.394
FOXE3	899	0.28
TIMP3	881	0.093
PIKFYVE	836	0.148
FTL	834	0.137
FOXC1	828	0.088
CRX	819	0.217

BEST1	815	0.081
RDH5	801	0.075
SLC45A2	786	0.201
CRYGC	768	0.066
VCAN	760	0.403
PAX6	760	0.48
PHOX2A	745	0.092
OPTN	717	0.088
GJA3	650	0.156
CHST6	649	0.065
KRT12	635	0.077
FBN1	633	0.319
OPN1MW	595	0.145
CA4	567	0.142
PRPF8	548	0.757
CFHR1	526	0.194
AIPL1	518	0.258
AIM1	516	0.197
CFHR3	510	0.167
ELOVL4	502	0.073

FBLN5	486	0.183
PLEKHA1	480	0.146
HMCN1	473	0.115
CNGA3	469	0.342
OPA3	469	0.092
RPE65	468	0.386
EFEMP1	456	0.408
ABCA4	443	0.347
KERA	438	0.204
TULP1	430	0.494
PDE6B	409	0.696
TACSTD2	405	0.386
PRPH	401	0.487
SAG	395	0.682
VSX1	385	0.216
ROM1	374	0.377
C1QTNF5	374	0.296
RHO	369	0.676
OPA1	367	0.597
RP1	348	0.673

CRYBA1	348	0.479
CRYGD	347	0.603
LAMB2	331	0.571
CACNA1F	324	0.557
CYP4V2	319	0.322
PRPH2	305	0.585
RGS9	291	0.523
FSCN2	281	0.44
IMPDH1	281	0.856
CRYAA	278	0.647
CRB1	277	0.77
CTDP1	260	0.789
PRPF31	260	0.897
GUCA1A	255	0.559
RPGR	235	0.773
CHM	234	0.792
COL8A2	221	0.489
RGS9BP	220	0.391
POLG	217	0.81
RP9	195	0.746

NYX	179	0.692
GJA8	167	0.795
HSF4	160	0.764
RLBP1	0	1
CNGB3	0	1
CERKL	0	1
KIF21A	0	1
OPN1LW	0	1
NHS	0	1
SPATA7	0	1
RPGRIP1	0	1

18. Genes related to psychiatric

Gene	Maximum interaction score	Permutation FDR
AKT1	997	0.221
BDNF	992	0.049
COMT	984	0.031
SLC6A3	963	0.044
DRD5	961	0.014
HCRT	954	0.081
DTNBP1	944	0.077

CYP2A6	917	0.209
HTR2A	907	0.13
DRD3	901	0.294
DRD4	899	0.687
MTHFR	854	0.156
HTT	818	0.271
PRODH	800	0.132
DLX2	742	0.133
SLC6A4	722	0.319
TPH2	685	0.234
RTN4R	582	0.221
CHI3L1	563	0.06
GABBR2	447	0.232
DISC1	416	0.866
CHRNA4	396	0.521
DAOA	384	0.448
NLGN3	321	0.452
DAO	279	0.598
KIAA0319	272	0.362
APOL2	191	0.585

APOL4	191	0.549
DYX1C1	0	1

19. Genes related to renal

Gene	Maximum interaction score	Permutation FDR
WT1	983	0.044
PKD1	980	0.02
HNF1B	947	0.044
FOXL2	929	0.019
AGTR1	928	0.094
INSL3	923	0.041
AVPR2	899	0.416
SCNN1B	887	0.077
SCNN1G	878	0.077
REN	838	0.239
COL4A5	836	0.068
ACE	834	0.176
NPHS1	832	0.274
CD2AP	818	0.372
TRPM6	817	0.042
NPHS2	815	0.164

TRPC6	800	0.254
SLC5A2	780	0.034
SLC22A12	716	0.102
SLC3A1	714	0.12
AGT	664	0.317
ACTN4	657	0.631
SLC12A3	656	0.147
SLC4A4	642	0.118
CD151	628	0.256
SLC4A5	587	0.066
NPHP3	563	0.104
CACNA1S	535	0.395
CLDN16	510	0.364
PKHD1	489	0.248
NPHP1	484	0.327
ATP6V0A4	480	0.418
INVS	405	0.388
DIAPH2	381	0.511
SLC7A9	358	0.299
COL4A3	298	0.7

CLCN5	296	0.656
UMOD	272	0.687
COL4A4	249	0.755
IQCB1	208	0.336
CTNS	151	0.586
NPHP4	0	1
ZNF365	0	1

20. Genes related to respiratory

Gene	Maximum interaction score	Permutation FDR
IFNGR1	999	0.017
IFNG	997	0.076
MMP1	995	0.032
IL12B	993	0.093
TNF	993	0.301
BDNF	992	0.049
ALOX5	983	0.043
TLR2	982	0.119
CISH	968	0.04
CFTR	966	0.108
IL13	964	0.145

HMOX1	962	0.045
CCL2	961	0.27
TIRAP	939	0.102
RET	932	0.284
MUC7	927	0.012
TGFB1	924	0.373
ADRB2	918	0.194
HLA-G	912	0.076
PLA2G7	910	0.137
CCL11	909	0.219
NPSR1	909	0.097
PTGDR	909	0.087
EDN3	899	0.615
MC3R	899	0.395
MUC5B	883	0.062
GDNF	863	0.383
FCGR2A	834	0.173
ASCL1	795	0.273
SERPINA1	784	0.409
SFTPB	736	0.208

SP110	716	0.05
FLCN	714	0.106
TSC2	686	0.532
CD209	670	0.346
TSC1	575	0.743
SCGB1A1	473	0.432
HNMT	470	0.464
SFTPA2	417	0.341
SLC11A1	377	0.546
PHF11	360	0.492
SCGB3A2	360	0.368
SFTPA1	337	0.532
PHOX2B	327	0.732
DNAH5	0	1

21. Genes related to skeletal

Gene	Maximum interaction score	Permutation FDR
BMPR1B	999	0.039
NOG	998	0.055
BMP2	997	0.088
COL2A1	990	0.028

RUNX2	989	0.08
SOST	987	0.008
FGFR3	983	0.099
SCT	979	0.07
IHH	963	0.082
GHR	959	0.118
SHH	956	0.362
GJA1	953	0.097
COMP	952	0.04
TGFB1	924	0.373
DLL3	916	0.052
CTSK	915	0.035
NPR2	899	0.475
FGFR1	899	0.393
FGFR2	878	0.33
EVC	871	0.019
SOX9	847	0.367
MSX1	804	0.264
IRF6	800	0.044
HOXD13	800	0.124

MSX2	764	0.304
PRG4	764	0.043
GDF5	730	0.205
ROR2	724	0.105
COL9A2	696	0.084
EVC2	694	0.055
FLNA	663	0.592
FBLN1	619	0.179
HOXD10	591	0.147
SRA1	585	0.331
MESP2	544	0.293
COL9A3	541	0.143
PAX9	527	0.429
WISP3	488	0.136
COL11A1	475	0.579
GHSR	450	0.287
TP63	433	0.744
LMBR1	424	0.191
THBS2	420	0.625
MATN3	408	0.31

EFNB1	385	0.685
ASPN	374	0.473
TBX4	358	0.282
SLC26A2	353	0.368
MNX1	351	0.879
OFD1	310	0.749
FLNB	296	0.634
TRAPPC2	278	0.3
CILP	196	0.382
DYM	0	1
LBR	0	1
SHOX	0	1

22. Genes related to unclassified

Gene	Maximum interaction score	Permutation FDR
LRP5	998	0.024
CFTR	966	0.108
ADH1B	963	0.063
PTEN	953	0.26
HTR2A	907	0.13
FSHR	899	0.456

FGFR2	878	0.33
GABRA2	504	0.102
SH3BP2	291	0.562
PLOD2	247	0.588
TAS2R16	0	1
RCBTB1	0	1
TSPYL1	0	1
